# Both systemic and local application of Granulocyte-colony stimulating factor (G-CSF) is neuroprotective after retinal ganglion cell axotomy

**DOI:** 10.1186/1471-2202-10-49

**Published:** 2009-05-14

**Authors:** Tobias Frank, Johannes CM Schlachetzki, Bettina Göricke, Katrin Meuer, Gundula Rohde, Gunnar PH Dietz, Mathias Bähr, Armin Schneider, Jochen H Weishaupt

**Affiliations:** 1 Department of Neurology, University Medical Center Göttingen, Robert-Koch-Strasse 40, 37075 Göttingen, Germany; 2 DFG-Research Center for Molecular Physiology of the Brain (CMPB), Humboldtallee 23, Göttingen, Germany; 3 H Lundbeck A/S, 2500 Valby, Denmark; 4 Sygnis Bioscience, Im Neuenheimer Feld 515, 69120 Heidelberg, Germany

## Abstract

**Background:**

The hematopoietic Granulocyte-Colony Stimulating Factor (G-CSF) plays a crucial role in controlling the number of neutrophil progenitor cells. Its function is mediated via the G-CSF receptor, which was recently found to be expressed also in the central nervous system. In addition, G-CSF provided neuroprotection in models of neuronal cell death. Here we used the retinal ganglion cell (RGC) axotomy model to compare effects of local and systemic application of neuroprotective molecules.

**Results:**

We found that the *G-CSF receptor *is robustly expressed by RGCs *in vivo *and *in vitro*. We thus evaluated G-CSF as a neuroprotectant for RGCs and found a dose-dependent neuroprotective effect of G-CSF on axotomized RGCs when given subcutaneously. As stem stell mobilization had previously been discussed as a possible contributor to the neuroprotective effects of G-CSF, we compared the local treatment of RGCs by injection of G-CSF into the vitreous body with systemic delivery by subcutaneous application. Both routes of application reduced retinal ganglion cell death to a comparable extent. Moreover, G-CSF enhanced the survival of immunopurified RGCs *in vitro*.

**Conclusion:**

We thus show that G-CSF neuroprotection is at least partially independent of potential systemic effects and provide further evidence that the clinically applicable G-CSF could become a treatment option for both neurodegenerative diseases and glaucoma.

## Background

Granulocyte-Colony Stimulating Factor (G-CSF) promotes the survival, differentiation and proliferation of cells of the neutrophilic lineage [1,2]. It belongs to the group of lineage-specific hematopoietic colony growth factors. However, G-CSF is able to pass the blood-brain-barrier despite of its high molecular weight [3]. Moreover, in the central nervous system (CNS) G-CSF is produced endogenously by neurons and its production can be enhanced by exogenous stimuli such as hypoxia [3]. The G-CSF receptor (G-CSFR), which is a single transmembrane protein, was recently found to be widely expressed in the CNS of adult rodents and humans [3]. Recombinant G-CSF was first approved by the FDA in 1991 for the treatment of patients with chemotherapy-induced neutropenia to lower the incidence of infections [4], and thus is a well established clinical drug with a favourable safety profile. Besides occasional bone pain, subcutaneous administration of G-CSF results in elevated white blood cell counts. However, long-term application of G-CSF for patients with chronic neutropenia was safe, particularly in regard to hematological malignancies [5-8].

In analogy to its anti-apoptotic effect in the hematopoietic system, G-CSF was recently found to reduce infarct volume and to improve functional recovery in rodent studies of acute and subacute stroke [3,9-18]. Consequently, several stroke trials on humans have been conducted (for review see: [19,20]). One small study with 10 patients showed reduced functional deficits after 6 months of treatment compared to the control group [21]. Recently, we corroborated the neuroprotective effect of G-CSF in the MPTP model of Parkinson's disease [22] and G-CSF was furthermore shown to attenuate striatal degeneration in a rodent model for Huntington's disease [23]. Moreover, elevated G-CSF levels were found in patients with amyotrophic lateral sclerosis, suggesting a possible endogenous neuroprotective effect of G-CSF for chronic neurodegenerative diseases [24,25]. In addition to direct neuroprotective and -regenerative effects mediated by G-CSFR expressed in the CNS, indirect mechanisms based on systemic G-CSF effects have also been discussed, such as immunomodulatory effects or the mobilization of bone marrow stem cells [18].

Here, we study the neuroprotective effect of G-CSF in the rat optic nerve (ON) transection model. ON axotomy leads to retrograde degeneration of 85% of the retinal ganglion cells (RGCs) within 14 days. In contrast to most other *in vivo *models for neurodegeneration, death of axotomized RGCs fulfils all criteria of apoptosis [26-29]. Besides classic morphological signs [28], axotomized RGCs show activation of caspase-3 and -9 [29-31] antagonistic regulation of Bax and Bcl-2 expression [26], downregulation of apoptosis-inhibiting activities of RAS/RAF/ERK or PI3K/Akt kinases [29,30,32] and protective effects of caspase inhibitors [29,31] and neurotrophins [29,30,33]. Due to the possibility of either systemic or intraocular application, the optic nerve transection paradigm permits to compare both routes of application for neuroprotective agents.

The ON axotomy model is thus an elegant *in vivo *paradigm for neurodegenerative diseases. In addition, with respect to the pressure-induced axonal damage found as a pathophysiological mechanism in glaucoma, it also reflects the selective degeneration of RGCs in this ophtalmological disease, which often continues despite adequate lowering of the intraocular pressure [34-36].

In summary, we found further evidence suggesting that G-CSF, a drug which has been in clinical use for almost twenty years with a very good safety record, is a potential clinical treatment option for both neurodegenerative diseases and glaucoma. Moreover, we show that in the *in vivo *model used here, local application of G-CSF to retinal ganglion cells is sufficient to induce neuroprotective effects independent of systemic mechanisms.

## Results

### G-CSF receptor expression in retinal ganglion cells *in vivo*

**Figure 1:**
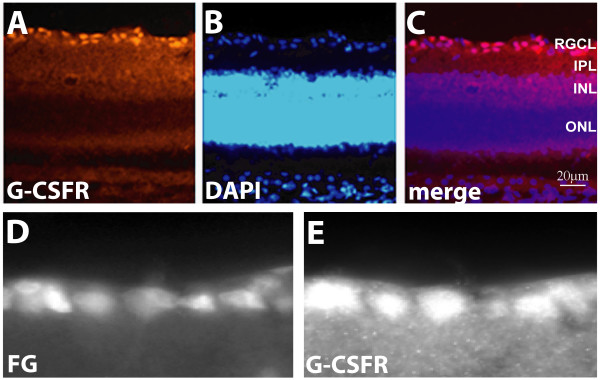
***G-CSF receptor *is expressed in retinal ganglion cells of the rat retina**. Robust G-CSFR immunoreactivity was detected in the RGC layer (A-C). RGCs were specifically labelled with the fluorescent tracer Fluorogold in (D). G-CSFR labelling in (E) shows that all RGCs expressed G-CSFR protein. Only faint G-CSFR immunoreactivity was detected in outer retinal layers (A-C). FG: Fluorogold; RGCL: retinal ganglion cell layer; IPL: inner plexiform layer; INL: inner nuclear layer; ONL: outer nuclear layer.

Using immunohistochemical staining with an antibody raised against the C-terminus of the G-CSFR we found expression in the retinal ganglion cell layer (Fig. 1A, C). DAPI counterstaining showed that immunoreactivity could be found on RGC somata, but no axonal or dendritic staining was observed. G-CSFR staining was largely restricted to the RGC layer, with only low immunoreactivity, hardly exceeding background levels, in the remaining retinal layers. Prelabelling of RGCs by injection of a retrograde tracer (Fluorogold; Fluorochrome) into the superior colliculus, resulting in retrograde, specific staining of RGCs, demonstrated that almost all RGCs were immunoreactive for G-CSFR protein (Fig. 1D). G-CSFR immunoreactivity remained unchanged 4 and 7 days after axotomy (data not shown).

### *In vivo *protective effects of systemic G-CSF application

**Figure 2:**
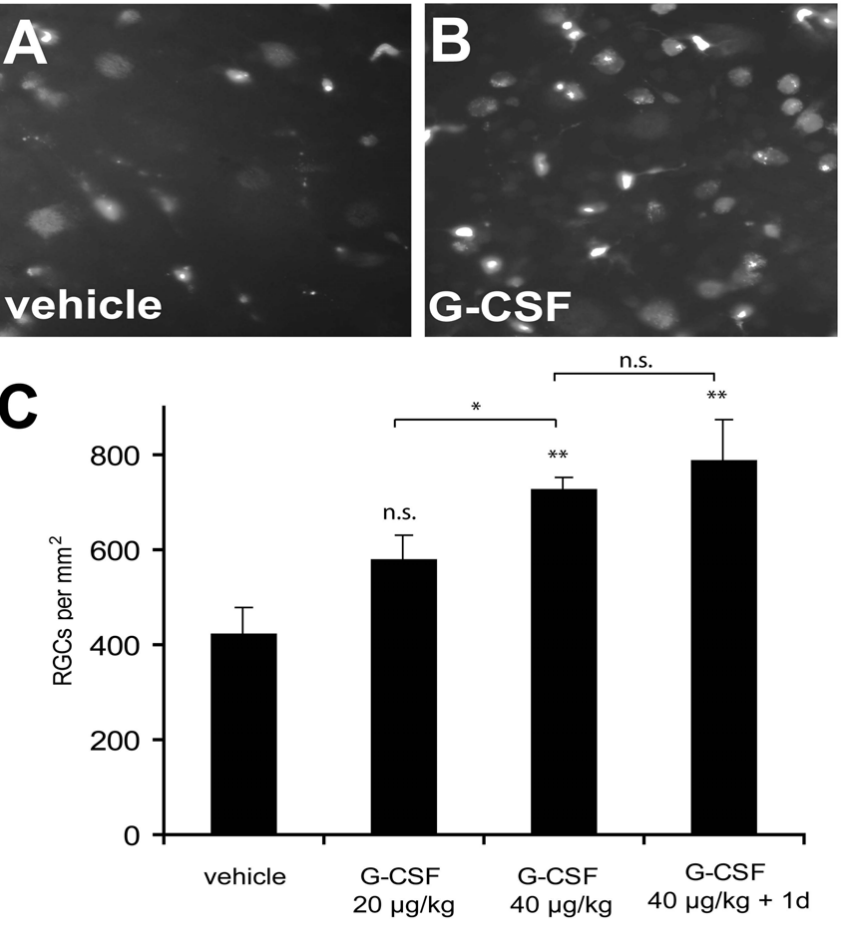
**G-CSF protects RGCs after optic nerve transection**. Subcutaneous daily injection of G-CSF (B, C) dose-dependently attenuated RGC apoptosis after optic nerve transection when compared to vehicle injection (A, C). 14 days after optic nerve transection reduction in RGC loss was statistically significant at a dose of 40 μg/kg bodyweight s.c. when the first injection was performed approximately 2 h after the optic nerve transection. Starting G-CSF injection one day before axotomy (40 μg/kg + 1 d) resulted in a slight, but non-significant increase in RGC numbers compared to treatment starting after the lesion. Panels (A) and (B) show an eccentricity at one half of the retinal radius. * p < 0.05 (20 vs. 40 μg/kg bodyweight); ** p < 0.01 (40 μg/kg or 40 μg/kg + 1 d vs. vehicle); n.s. = not significant. Data are given as mean ± S.E.M.

Based on the positive G-CSFR immunoreactivity on RGCs, we asked whether G-CSF is able to prevent RGC degeneration following axotomy due to optic nerve transection. Similar to earlier data [37,38], we observed 420.6 ± 58.4 surviving RGCs/mm^2 ^14 days after optic nerve transection and daily subcutaneous vehicle injection (see material and methods; Fig. 2A, C). Based on G-CSF dosing in previous *in vivo *neuroprotection experiments [3,22], we then applied a daily dose of 20 or 40 μg/kg bodyweight (BW) G-CSF subcutaneously, with the first injection approximately 2 h after axotomy. While application of 20 μg/kg BW showed a non-significantly enhanced survival of RGCs (578.9 ± 52.0 surviving RGCs/mm^2^; table 1, Fig. 2B), a dose of 40 μg/kg BW G-CSF s.c. resulted in a highly significant neuroprotective effect (742.3 ± 25.8 RGCs/mm^2^; p < 0.001; table 1, Fig. 2B, C).

**Table 1:** Summary of *in vivo *RGC counts. Systemic and topical application of G-CSF or vehicle.

**Experimental Group**	**Animal**	**RGCs per mm^2^**	**Mean**	**S.E.M.**
axotomy + vehicle injection s.c.	1	360		
	
	2	348		
	
	3	594		
	
	4	379	420.6	58.4

axotomy + vehicle injection i.o.	1	443		
	
	2	390		
	
	3	378		
	
	4	435		
	
	5	365	402.2	15.5

axotomy + G-CSF 20 μg/kg BW s.c. daily	1	669		
	
	2	442		
	
	3	554		
	
	4	649	578.9	52.0

axotomy + G-CSF 40 μg/kg BW s.c. daily	1	729		
	
	2	637		
	
	3	778		
	
	4	704		
	
	5	773	742.3	25.8

axotomy + G-CSF 40 μg/kg BW s.c. daily (start 1 d before surgery)	1	641		
	
	2	876		
	
	3	696		
	
	4	872	771.3	60.3

axotomy + G-CSF 500 ng i.o. on days 0, 4, 7 and 10 after axotomy	1	638		
	
	2	613		
	
	3	773		
	
	4	703	681.8	35.8

Cell numbers are given as mean ± S.E.M. s.c.: subcutaneous; i.o.: intraocular; BW: bodyweight.

Next, we tested whether additional pre-treatment with 40 μg/kg BW G-CSF s.c. starting one day before axotomy could further increase RGC survival. We found that this pre-treatment resulted in a slight, but non-significant increase in RGC numbers compared to treatment starting after the lesion (771.3 ± 60.3 RGCs/mm^2^; table 1).

### G-CSF enhances survival of immunopurified RGCs deprived of neurotrophic factors

**Figure 3:**
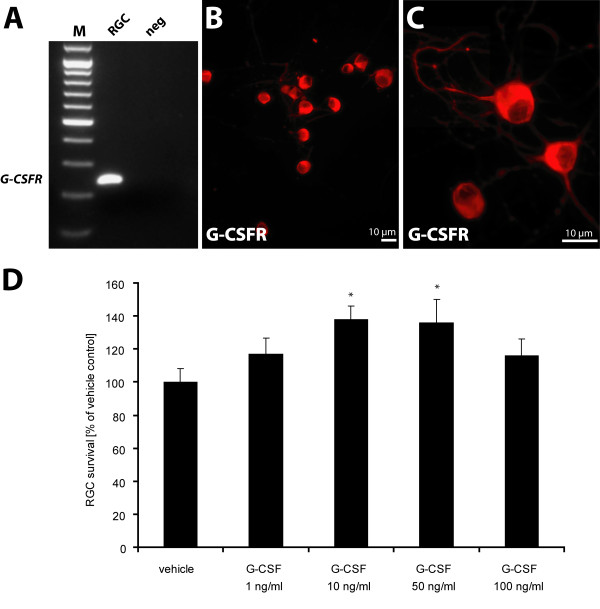
**G-CSF promotes survival of immunopurified rat RGCs after neurotrophic factor deprivation**. RGCs from 6- to 8-day old rat pups were immunopurified and cultured under full neurotrophic support for 24 hours before neurotrophins were withdrawn. (A) RT-PCR from primary RGCs demonstrates *G-CSFR *expression at the mRNA level. The resulting PCR product has the expected 233 bp size. (B, C) G-CSFR immunocytochemistry on primary RGCs demonstrates receptor expression at the protein level. (C) shows higher power magnification. (D) RGCs were cultured under full neurotrophic support for 24 h. Cells were then deprived of neurotrophins, and at the same time G-CSF (1, 10, 50 or 100 ng/ml) or vehicle was added. The number of surviving RGCs was determined by MTT staining after an additional 2 days *in vitro*. A bell-shaped dose-response curve is observed, demonstrating significantly enhanced survival of RGCs compared with the vehicle-treated control cultures at a concentration of 10 and 50 ng/ml. * p < 0.05 (compared with vehicle). Data are given as mean ± S.E.M.

Although distinct neuronal subtypes express the G-CSFR, indirect mechanisms based on systemic G-CSF effects have also been discussed as a mechanism for G-CSF neuroprotection. Therefore, in order to find further evidence for a direct action of G-CSF on RGCs, we employed immunopurified rat RGC cultures. Immunopurified rat RGCs provide a well-established *in vitro *model for studies on retinal ganglion cell death and apoptosis of CNS neurons in general. Moreover, they share important aspects of cell survival mechanisms with in situ RGCs [39-41]. A two-step immunopurification protocol [42] was used to purify RGCs to near homogeneity. Subsequently the cells were cultured in serum-free medium, supplemented with the neurotrophic factors forskolin, human BDNF, CNTF, and insulin (see material and methods). G-CSFR expression in purified RGCs was confirmed by RT-PCR and immunocytochemistry (Fig. 3A–C). After 1 day in culture, neurotrophins were withdrawn, and the number of surviving RGCs was determined after an additional 2 d *in vitro*. When G-CSF was added concomitantly with the neurotrophin deprivation, survival of immunopurified RGCs was enhanced significantly (Fig. 3D), suggesting a direct neuroprotective effect of G-CSF on RGCs.

### Topical application is sufficient for G-CSF-mediated neuroprotection *in vivo*

**Figure 4:**
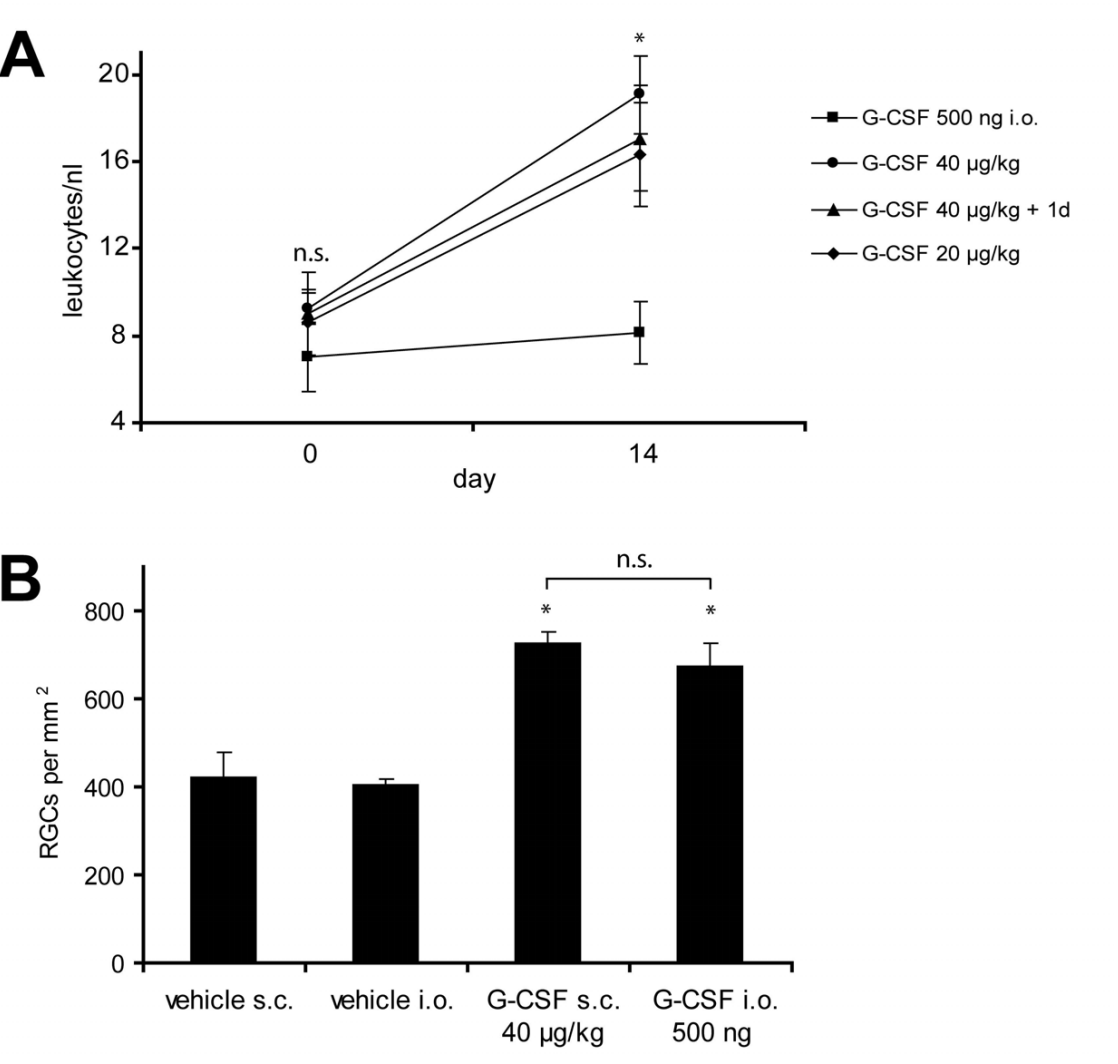
**Topical application by intravitreal injection is sufficient for G-CSF neuroprotection on retinal ganglion cells**. G-CSF was given either systemically by daily subcutaneous injection (20 or 40 μg/kg bodyweight per injection), or by direct application to RGCs via intravitreal injection (500 ng in 2 μl per injection on day 0, 4, 7 and 10 after axotomy; first injection 2 h after axotomy). Leukocyte counts were determined one day before drug administration and after 14 days of treatment (n = 8 per group). Subcutaneous application resulted in the expected leukocytosis, while we could confirm that the intraocular G-CSF injection did not influence leukocyte counts (A). RGC survival was then determined accordingly after subcutaneous or intraocular G-CSF application (B). Both protocols lead to a comparable protective effect on axotomized RGCs *in vivo*. * p < 0.05 when compared to respective vehicle control; n.s. = not significant; i.o.: intraocular (intravitreal) injection.

Our *in vivo *optic nerve transection paradigm offers the advantage that protective compounds can be applied locally to the neuronal population of interest by direct intravitreal injection. We thus performed intravitreal injections of vehicle or G-CSF at days 0, 4, 7 and 10 after axotomy (500 ng G-CSF per injection in a volume of 2 μl, first injection approximately 2 h after the axotomy) in order to test whether systemic effects are required for G-CSF neuroprotection *in vivo*. We confirmed that, in contrast to the expected higher leukocyte counts after subcutaneous G-CSF application, the intraocular injection protocol did not increase blood leukocyte numbers (Fig. 4A). In agreement with the observed protective effect of G-CSF on purified RGCs in culture, we found that direct intraocular application of G-CSF was sufficient for neuroprotective effects (681.8 ± 35.8 surviving RGCs/mm^2^; p < 0.05 compared to vehicle control), and did not significantly differ from the effect of 40 μg/kg BW G-CSF given subcutaneously (Fig. 4B; table 1).

## Discussion

G-CSFR expression on neurons has been demonstrated few years ago [11], and G-CSFR expression by neuronal and non-neuronal cells has recently been identified in numerous regions of the brain [3,22]. Besides other cell types, it was lately found to be expressed by neuronal populations that are subject to cell death in neurodegenerative diseases, e.g. motoneurons [[24,25], own unpublished observation] or dopaminergic neurons in the substantia nigra [22,43].

Similar to the expression pattern of the erythropoietin receptor (EPOR), another hematopoietic cytokine with neuroprotective action, we show for the first time constitutive expression of the G-CSFR on the somata of RGCs. In contrast to the EPOR [38] we could not find G-CSFR expression on the dendrites of the RGCs *in vivo*. Our finding that the G-CSFR is expressed by RGCs prompted us to investigate possible neuroprotective effects of G-CSF on RGCs. We demonstrate profound neuroprotection against axotomy-induced RGC death through G-CSF. 40 μg/kg BW s.c. lead to a significant attenuation of RGC cell loss after optic nerve transection even when G-CSF was given after lesioning. The mode of RGC death in the model we used was shown to homogenously exhibit classical features of apoptosis [26-31]. Thus, based on the large body of evidence showing almost exclusively apoptotic cell death in this paradigm, the substantial reduction in RGC death can be explained by an anti-apoptotic activity of G-CSF. An impact of G-CSF on other confounding parameters, e.g. on the retrograde transport of Fluorogold, is theoretically possible. However, retrograde Fluorogold labelling of RGCs is already completed two days after axotomy [44], while rats were processed 14 days after axotomy in our experiments. Moreover, Fluorogold was proven to be a reliable marker for RGC survival that correlates well with markers for apoptosis, also under treatment with protective neurotrophins or cytokines in several studies [30,33]. Finally, our data from purified RGC primary cultures further support an anti-apoptotic effect of G-CSF independent from retrograde axonal dye transport.

It has been postulated that G-CSF may confer an improved outcome in animal stroke models by bone marrow stem cell mobilization and migration to the lesion site, followed by neuronal differentiation [12]. Additional systemic mechanisms could be postulated, as G-CSF has prominent systemic anti-inflammatory properties [45] that might contribute to its neuroprotective action in this model. In the experimental autoimmune encephalitis (EAE) model of multiple sclerosis, G-CSF was found to reduce T-cell-recruitment to the CNS and protected from further inflammation [46]. Moreover, G-CSF could be neuroprotective by indirect mechanisms, e.g. inducing the release of neurotrophic factors from glial cells [47]. However, G-CSF might also act directly on neural cells after systemic application as it passes the intact blood brain barrier [3]. Furthermore, we [[22], this study] and others [3,11] found the G-CSFR expressed on neuronal target cells, and neuroprotection was described in cultures of neuronal cell lines free of non-neuronal cells, suggesting a direct protective action of G-CSF within the nervous system [3,22]. Our data presented here show that local application of G-CSF to the retina is sufficient to induce neuroprotection *in vivo*. Moreover, the results from our *in vitro *experiments using primary cultures of immunopurified RGCs argue in favour of a direct inhibition of neuron-specific apoptotic pathways by activation of neuronal G-CSFR. Nevertheless, this does not exclude an additional contribution of the above mentioned indirect mechanisms, e.g. glial cell-derived neurotrophic factors. Similarly, potential systemic effects that might partially contribute to G-CSF neuroprotection beyond its local activity can not be excluded at this point. However, the finding of substantial CNS neuroprotection after local injection of G-CSF could become of clinical relevance if, in order to avoid potential unwanted systemic effects of G-CSF, intracerebral or intrathecal administration will be tested in future treatment studies for neurodegenerative diseases.

In contrast to most other neurotrophic molecules, G-CSF is already used in clinical practice for many years to treat neutropenic patients, e.g. after chemotherapy or in cases of severe congenital neutropenia. Especially the latter application, which often requires G-CSF treatment for many years, yielded valuable safety data showing that also long-term treatment with G-CSF is principally possible [7]. Thus, G-CSF could be immediately transferred to clinical studies. Nevertheless, a safety record for the chronic treatment of neurodegenerative diseases with G-CSF, also with respective dose-finding studies, will have to be performed in phase IIa/b studies.

As already outlined in the introduction, cell death in our optic nerve transection model displays the properties of classical neuronal apoptosis. Therefore, it provides an elegant opportunity to study potential new treatment concepts for neurodegenerative diseases in an *in vivo *model. In addition, our model replicates important steps in the pathologic course of glaucoma, because axonal lesions due to increased ocular tension are thought to induce retrograde RGC death and vision loss [48-50]. Therefore, besides its potential usefulness for the treatment of neurodegenerative diseases, G-CSF may also be a powerful add-on drug to stop RGC degeneration and to stop or slow down vision loss, especially since lowering the intraocular pressure does not necessarily halt the continuous degeneration of RGCs. Further, autoantibody-induced RGC apoptosis is a possible mechanism of autoimmune retinopathy in cancer patients and may represent another potential therapeutic application for G-CSF in ophthalmology [51].

## Conclusion

In summary, we provide further support for the use of G-CSF as a neuroprotective molecule that could be transferred to clinical application in the near future. We demonstrate that G-CSF exerts neuroprotection after local delivery to CNS neurons *in vivo *and may also become a valuable therapeutic option in ophthalmology.

## Methods

### Transection of the optic nerve, retrograde labelling, tissue processing, and cell counting

Adult female Sprague-Dawley rats (200–250 g) were purchased from Charles River, Wiga, Germany. Animals were housed in plastic cages under standard laboratory conditions. For a more detailed review of the surgery and tissue processing see Kermer et al. [32] Briefly, animals were anaesthetized by intraperitoneal injection of chloral hydrate (0.42 g/kg BW). The optic nerve (ON) was transected approximately 2 mm from the posterior eye pole without damaging the retinal blood supply. In order to label RGCs, a small piece of gel foam soaked in Fluorogold (FG, Fluorochrome, CO, USA) was placed at the ocular stump of the axotomized ON. Alternatively, specific Fluorogold staining was achieved by injection of the tracer into the superior colliculus. On day 14, animals were sacrificed by injecting an overdose of chloral hydrate. The eyes were removed and dissected, removing cornea, lens, and vitreous body. The eye bulb was then fixed for 20 minutes in 4% PFA, followed by separation of the retina from the eye bulb. This was followed by flat-mounting the retina on gelatine-coated glass slides incising the tissue to four retinal quadrants. RGCs were examined under a fluorescence microscope (Axiovert 35; Carl Zeiss Meditec) using a UV filter (365/420 nm) to detect Fluorogold fluorescence. The number of FG – positive RGCs was determined by counting them in 12 distinct areas of 62,500 μm^2 ^each (three areas per retinal quadrant at three different eccentricities of one sixth, one half, five sixths of the retinal radius). Cell counts were performed by two different blinded observers and the average of their cell counts was calculated. Overestimation of RGC density by retinal shrinkage due to i.o. injection was excluded before [33]. Animal experiments were approved by appropriate German authorities (Bezirksregierung Braunschweig).

### Drug administration

For intravitreal drug administration, animals were anesthetized with diethyl ether. Via a glass microelectrode with a tip diameter of 30 μm, G-CSF (Neupogen^®^, AMGEN, Thousand Oaks, USA) or respective vehicle (250 mM sorbitol, 0.004% Tween-80 and 10 mM sodium acetate buffer (pH 4,0))was injected into the vitreous space (500 ng in 2 μl injection volume), puncturing the eye behind the cornea-sclera junction and carefully avoiding the lens as described [32]. Injections were performed on days 0, 4, 7, and 10 after axotomy. Alternatively, G-CSF was given subcutaneously once daily at a dose of 20 or 40 μg/kg bodyweight, or the respective vehicle was injected.

### Immunohistochemistry

For immunohistochemical experiments, animals were killed 3 days after surgery with an overdose of chloral hydrate. The eyes were dissected and eye cups immediately fixed without cornea and lens for 1 hour in 4% PFA in PBS at 4°C. Eye cups were immersed in 30% sucrose in PBS overnight at 4°C and frozen in cutting compound (Tissue-tek; Sakura Finetek, Torrance, CA) with liquid nitrogen. Cryostat-cut sections (16 μm) were collected on slides (Superfrost; Fisher Scientific, Pittsburgh, PA), dried at 37°C, and stored at -20°C. Slides were then dried for 1 hour at 37°C, cryosections were incubated in 10% normal goat serum (NGS, PAA) in PBS containing 0,3% Triton X-100 (Roth, Karlsruhe, Germany), to block non-specific binding. Sections were then washed three times in PBS and incubated with primary antibody at 4°C overnight. Immunoreactivity was visualized by incubating the sections with a secondary fluorescent antibody or with a biotinylated secondary antibody after washing three times with PBS followed by applying avidin-biotin-complex (ABC-Elite; Vector Laboratories, CA, USA). Finally, sections were cover slipped (Fluka, Sigma-Aldrich, Buchs, Switzerland). Primary antibody was directed against G-CSFR (1:100, Santa Cruz Biotechnology, Santa Cruz, CA). Goat anti-rabbit Cy3-conjugated IgG served as secondary antibody (Dianova, Hamburg, Germany). Immunofluorescence and DAB staining was imaged with an Axioplan microscope (Carl Zeiss, Meditec).

### Immunopanning and immunocytochemistry of RGCs

*In vitro *experiments using immunopurified retinal ganglion cell culture were prepared as described previously [42]. Briefly, for immunopurified rat RGC culture, rat pups were killed on postnatal day 8. Serum-free culture medium was used (Neurobasal; Invitrogen-Gibco, Eggenstein, Germany) supplemented with glutamine, cysteine, pyruvate, triiodothyronine, B-27 supplement and Sato (BSA, transferrine, progesterone, putrescine, sodium selenite). During the first 24 hours, RGCs were additionally incubated with saturated concentrations of forskolin, human BDNF, ciliary neurotrophic factor (CNTF), and insulin. The culture was withdrawn from neurotrophins by change of medium on culture day 1 and incubated with vehicle or the indicated concentration of G-CSF. G-CSF was diluted in cell culture medium without neurotrophins to the respective final concentration in the cell culture well.

The RGC survival rate was determined by a 3-(4,5-dimethylthiazol-2-yl)-2,5-diphenyltetrazolium bromide (MTT) cell survival assay. MTT (5 mg/ml) was added to culture wells (1:10) at culture day 3 and incubated at 37°C for 1 hour. Viability of RGCs was assessed by counting six fields within a culture well using a 20× objective by a blinded observer. RGCs showing dense blue staining of cell bodies were considered to be MTT-positive. Survival of RGCs in the different culture conditions was calculated as the mean number of MTT-positive RGCs/mm^2 ^in three wells per experiment and per experimental condition on day 3. Results are expressed as the percentage of additional survival compared to neurotrophin-deprived control RGCs on day 3.

For immunocytochemistry primary RGCs were seeded on 24 well plates (5000/well) and fixed with PFA 4% after 3 days. Blocking was performed with 10% NGS for 30 min. Primary antibody (rabbit polyclonal antibody, SantaCruz, sc-694) was diluted in 2% NGS 1:50 and incubated overnight at 4°C. As secondary antibody, a goat-anti-rabbit Cy3 (Dianova, diluted 1:250) that was incubated for 1 h at room temperature was used. Coverslips were mounted on object slides with moviol.

### Reverse transcription and G-CSF receptor PCR from primary RGCs

RNA was prepared from primary RGCs by Qiagen RNA-Easy columns. Reverse transcription was performed using oligo dT primers and Transcriptor reverse transcriptase (Roche). For detection of the G-CSF receptor [GenBank: NM_001106685] the following primers were used: ratGCSFR-frag32s (CCATTGTCCATCTTGGGGATC) and rat-GCSFR-frag265as (CCTGGAAGCTGTTGTTCCATG) with an expected product length of 233 bp. Cycling conditions were as follows: 5 minutes at 94°C (only first cycle); 30 seconds at 94°C, 1 min at 66°C, 40 seconds at 72°C for 50 cycles; final elongation for 5 min at 72°.

### Statistics

Data were expressed as the mean ± SEM. Statistical significance was assessed applying one-way ANOVA followed by the Bonferroni's post hoc test (comparing RGC survival).

## Authors' contributions

TF performed cell counting, immunocytochemistry, RT-PCR, interpretation of results and prepared the figures and drafted the manuscript. JCMS performed cell counting and immunohistochemistry and drafted the manuscript. BG was responsible for surgery of rats. KM helped with immunohistochemistry and surgery. GR prepared RGC cultures. GPHD helped with RGC culture and data mining. MB and AS contributed to the experimental design and helped to draft the manuscript. JHW had the idea for the project, outlined the experimental design, interpreted results and helped with manuscript writing. He was also responsible for surgery, cell counting and immunohistochemistry. All authors read and approved the final manuscript.
